# A Multimodal Affinity Fusion Network for Predicting the Survival of Breast Cancer Patients

**DOI:** 10.3389/fgene.2021.709027

**Published:** 2021-08-20

**Authors:** Weizhou Guo, Wenbin Liang, Qingchun Deng, Xianchun Zou

**Affiliations:** ^1^College of Computer and Information Science, Southwest University, Chongqing, China; ^2^Key Laboratory of Luminescence Analysis and Molecular Sensing, Ministry of Education, College of Chemistry and Chemical Engineering, Southwest University, Chongqing, China; ^3^Department of Gynecology, The Second Affiliated Hospital of Hainan Medical University, Hainan, China

**Keywords:** deep learning, cancer survival prediction, self-attention mechanism, affinity network, multimodal data fusion

## Abstract

Accurate survival prediction of breast cancer holds significant meaning for improving patient care. Approaches using multiple heterogeneous modalities such as gene expression, copy number alteration, and clinical data have showed significant advantages over those with only one modality for patient survival prediction. However, existing survival prediction methods tend to ignore the structured information between patients and multimodal data. We propose a multimodal data fusion model based on a novel multimodal affinity fusion network (MAFN) for survival prediction of breast cancer by integrating gene expression, copy number alteration, and clinical data. First, a stack-based shallow self-attention network is utilized to guide the amplification of tiny lesion regions on the original data, which locates and enhances the survival-related features. Then, an affinity fusion module is proposed to map the structured information between patients and multimodal data. The module endows the network with a stronger fusion feature representation and discrimination capability. Finally, the fusion feature embedding and a specific feature embedding from a triple modal network are fused to make the classification of long-term survival or short-term survival for each patient. As expected, the evaluation results on comprehensive performance indicate that MAFN achieves better predictive performance than existing methods. Additionally, our method can be extended to the survival prediction of other cancer diseases, providing a new strategy for other diseases prognosis.

## 1. Introduction

Breast cancer is the second leading cause of death from cancer in women (Bray et al., 2018; McKinney et al., 2020). According to the estimation by American Cancer Society, there are more than 2.3 million new cases of invasive breast cancer diagnosed among females and approximately 685,000 cancer deaths in 2020 (Sung et al., 2021). Accurate survival prediction is an important goal in the prognosis of breast cancer patients, because it can aid physicians make informed decisions and further guide appropriate therapies (Sun et al., 2007). However, the high-dimensional nature of the multimodal data makes it hard for physicians to manually interpret these data (Cheerla and Gevaert, 2019). Considering this situation, it is urgent to develop computational methods to provide efficient and accurate survival prediction (Cardoso et al., 2019; Zhu et al., 2020).

The goal of cancer survival prediction is to predict whether and when an event (i.e., patient death) will occur within a given time period (Gao et al., 2020). In recent years, a considerable amount of work has been done to predict the survival of breast cancer patients by applying statistical or machine learning methods to single-modular data, especially gene expression data (Wang et al., 2005; Nguyen et al., 2013). For example, Van De Vijver et al. (2002) used multivariate analysis on gene expression data to identify 70 gene prognostic signatures. Xu et al. (2012) utilized support vector machine (SVM) to select key features from gene expression data for the survival prediction of breast cancer. However, these methods solely based on gene expression data still leave room for improvement, albeit with high performance (Alizadeh et al., 2015; Lovly et al., 2016). Especially with the advancement of next-generation sequencing technologies, there is a tremendous amount of multimodal data being generated, such as gene expression data, clinical data, and copy number alteration (CNA) (Peng et al., 2005). These data are extensively providing information for the diagnosis of cancer.

Recently, researchers have begun to integrate multimodal data to predict survival of cancer patients. For example, Sun et al. (2018), for the first time, developed a multimodal deep neural network that uses decision fusion to integrate multimodal data. Cheerla and Gevaert (2019) proposed an unsupervised encoder to compress clinical data, mRNA expression data, microRNA expression data, and histopathology whole slide images (WSI) into single feature vectors for each patient; these feature vectors were then aggregated to predict patient survival. Nikhilanand et al. (Arya and Saha, 2020) introduced a STACKED_RF method based on a stacked integrated framework combined with random forest in multimodal data. These results show that better performances can be achieved with multimodal data. Although many efforts have been dedicated to integrating multimodal data for cancer survival prediction, it remains a challenging task. First, features associated with survival only exist in tiny lesion regions, thus the feature embedding extracted from multimodal data might be dominated by excessive irrelevant features in normal areas and yield restrained classification performance. Second, there is abundant structured information between patients and multimodal data.

In this paper, we address the above two challenges by proposing a novel MAFN for integrating gene expression, CNA, and clinical data to predict survival of breast cancer patients. Our MAFN framework includes attention module, affinity fusion module, and deep neural networks (DNN) module. In order to capture critical features in the tiny lesion regions, we utilized attention mechanism to adaptively localize and enhance the features associated with the supervised target while suppressing background noise. However, the traditional attention mechanism (Gao et al., 2018; Chen et al., 2019; Uddin et al., 2020) is not compatible with the need for multimodal data, because its ignorance of the heterogeneity of multimodal data would lead to great weight assigned to a few features (Gui et al., 2019). Therefore, we applied a shallow attention net to each feature, which can effectively extract key information from multimodal data, fully taking the distinction and uniformity of heterogeneous data into account. Additionally, we utilized affinity fusion module to calculate fusion feature representation and to model complex intra-modality and inter-modality relations with the knowledge of structured information between patients and multimodal data. Meanwhile, the DNN module was used to compensate the lack of single-modality specific information on fusion features. The main contributions of this paper can be summarized as follows:

1. An attention module is proposed to adaptively localize and enhance the features associated with survival. By providing a shallow attention network for each feature, mechanism alleviates the problem of few features with great weight caused by data heterogeneity.
2. A novel feature fusion method is proposed, which constructs an affinity network to fuse multimodal data more effectively.
3. A multimodal data fusion method based on affinity network (MAFN) is proposed by integrating gene expression data, CNA data, and clinical data. We validate the effectiveness of MAFN and suggest building blocks on four exposed datasets. The experimental results show that MAFN performs better compared with existing research methods to the best of our knowledge (Jefferson et al., 1997; Xu et al., 2012; Nguyen et al., 2013; Sun et al., 2018; Chen et al., 2019; Arya and Saha, 2020).

The rest of this paper is organized as follows: section 2 presents the details of our proposed method and datasets. Furthermore, the experimental results are discussed in section 3 and some conclusions are drawn in section 4.

## 2. Materials and Methods

### 2.1. Materials

In this study, we used 4 independent breast cancer datasets, containing in total 3,380 samples (Table 1). We downloaded METABRIC dataset from cBioPortal (Curtis et al., 2012), and other datasets from the University of California Santa Cruz (UCSC) cancer browser website (Goldman et al., 2018). The downloaded datasets consist of three sub-data, including gene expression data, CNA data, and clinical data. We used these datasets in the following two steps. The first step was to obtain the labels of survival-risk classes from the clinical data of each dataset. Similar to the previous work by Khademi and Nedialkov (2015), each sample was labeled as a good sample if the patient survive more than 5 years, and labeled as a poor sample if the patient did not survive more than 5 years. The second was to randomly divide each dataset into three groups, 80% of the samples used as training set, 10% used as test set, and the remaining 10% used as verification set.

**Table 1:** Summary of breast cancer datasets.

**Name**	**Total samples**	**Poor samples**	**Good samples**	**Reference**
METABRIC	1,980	491	1,489	Pereira et al., 2016
TCGA-BRCA	1,056	813	243	The Cancer Genome Atlas
GSE8757	171	25	146	Chin et al., 2006
GSE69035	173	63	110	Zhang et al., 2009
Total	3,380	1,392	1,988	This work

### 2.2. Data Preprocessing

The preprocessing strategies for three sub-data (i.e., gene expression data, CNA data, and clinical data) were implemented as below. First, we matched the sample labels shared among three sub-data. Second, we filtered out the samples that have feature missing values (NA) of more than 20% and features with missing values (NA) in more than 10% samples for each sub-data. Then we estimated the remaining missing values using the k-nearest neighbor algorithm (Troyanskaya et al., 2001; Ding et al., 2016). Third, the gene expression features were standardized and further processed into three categories according to two thresholds (Sun et al., 2018): under-expression (–1), normal expression (0), and over-expression (1). These thresholds depend on the variance of the gene. A gene with high variance would receive a higher threshold than a gene with low variance. For CNA data, we directly utilized the original data with five discrete values: homozygous deletion (–2); hemizygous deletion (–1); neutral/no change (0); gain (1); high level amplification (2). For clinical data, non-numerical clinical data were digitized by one-hot encoding.

Feature Selection: The “curse of dimensionality” is a typical problem when using multimodal data (Tan et al., 2015; Nguyen et al., 2020). For example, the gene expression data and CNA data in METABRIC dataset contain 24,369 and 22,545 genes, respectively. Modified mRMR (MRMR) (Peng et al., 2005) is one of the common dimensionality reduction algorithms in a wide range of applications. Hence, we applied modified mRMR method (fast-MRMR) (Ramírez-Gallego et al., 2017) to select features from the original dataset without significant loss of information. Similar to the previous work (Zhang et al., 2016; Sun et al., 2018), we used the area under curve (AUC) value as the criteria to evaluate the performance of the features. In detail, we roughly searched the best N features from 200 to 600 with a step size of 100 (Table 2).

**Table 2:** The number of feature after feature selection.

**Name**	**Gene expression data**	**CNA**	**Clinical data**
METABRIC	400	200	25
TCGA-BRCA	400	200	23
GSE8757	300	300	17
GSE69035	400	300	21

### 2.3. Methods

In this section, we introduce the detailed design of MAFN for predicting the survival of breast cancer patients. The goal of MAFN is to distinguish between poor samples and good samples. The multimodal data as input consists of gene expression data, CNA data, and clinical data. It is expressed as follows:

(1) $$\begin{array}{l} X=\left\{X_{g},X_{c},X_{clin}\right\}\in R^{N\times d} \end{array}$$

where *d* = (*m* + *n* + *c*), and m, n, and c represent the dimension of the gene expression data, the CNA data, and the clinical data, respectively, and N is the number of patients.

#### 2.3.1. Attention Module

In order to adaptively localize and enhance the features associated with survival, we used an attention mechanism framework to guide our method. Previous attention mechanism-based studies for cancer survival prediction generate feature weights uniformly on all feature dimensions (Gao et al., 2018; Chen et al., 2019), which may not be a good choice for heterogeneous data sources. Because the heterogeneity of data results in few features of a single modal assigned with relatively large weights and the loss of details in the feature set. We argue that different modalities of one patient together reflect the patient's survival risk. To address this issue, in MAFN we propose attention module that is inspired by recent achievement in self-attention (Gui et al., 2019). We used a dedicated shallow Attention Net for each feature in X, alleviating the problem of data heterogeneity. The module consists of three main parts: (1) embedding layer; (2) Attention Net; and (3) sigmoid normalization.

First, an embedding layer network was used to extract the intrinsic information (denoted as E) from the raw input $X\in R^{N\times d}$ and eliminate noise. At the same time, the gene expression data and CNA data from large sparse domain were mapped to the dense matrix. The embedding layer is calculated as follows:

(2) $$\begin{array}{l} E=\sigma \left(X^{T}W_{E}+b_{E}\right) \end{array}$$

where *W*_*E*_ and *b*_*E*_ are trainable weight matrices, and σ(.) denotes activation function Relu(). The size of the embedding layer is *E*_*N*_, which is generally smaller than the size of the original input feature. In this process, the major part of information was retained, while some redundant information was discarded on the contrary.

**Figure 1:**
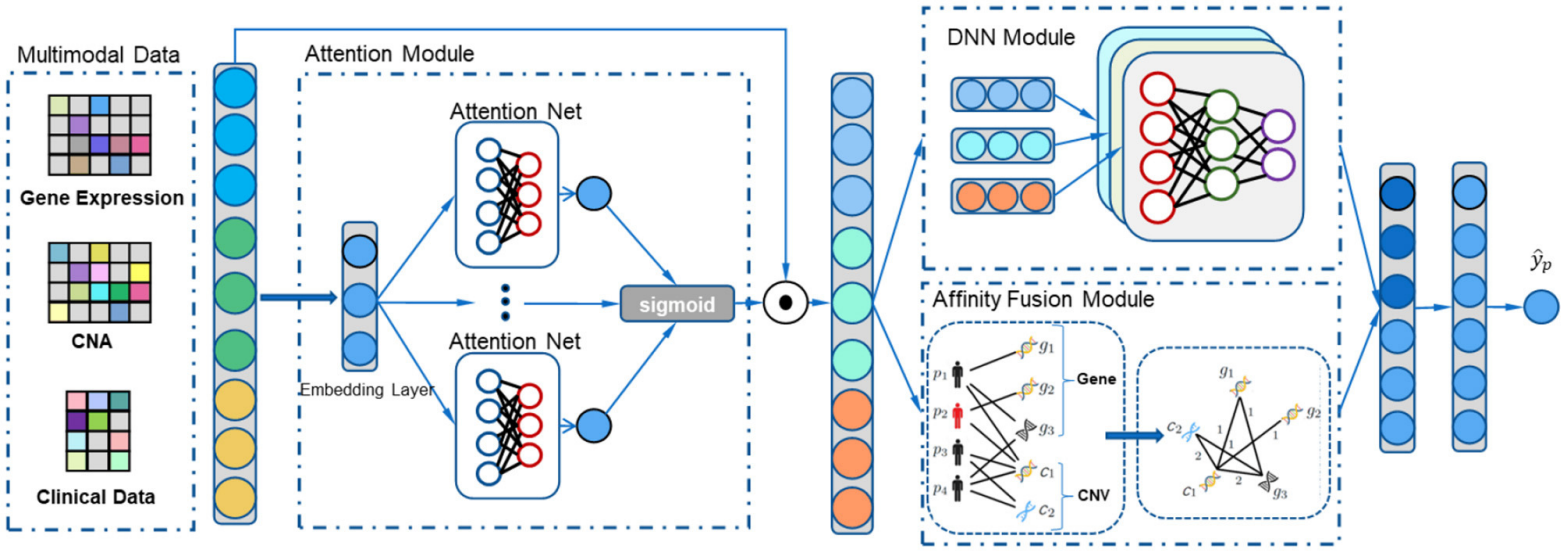
The overall process of our multimodal affinity fusion network (MAFN) model for the breast cancer survival prediction. It mainly includes three parts: (1) attention module adaptively localize and enhance the features associated with survival; (2) affinity fusion module extracts multimodal data fusion features; (3) DNN module extracts each modal specific feature.

Second, a stack-based shallow self-attention network was used to seek the probability distribution for each feature (Figure 1, attention module), respectively. Using $E\in R^{N\times E_{N}}$ extracted by the embedding layer as the input of each Attention Net, the *k*th feature's Attention Net (L layer) output weight *p*^*k*^ is then given by:

(3) $$\begin{array}{l} p^{k}=F_{1\to L}\left(E\right)=f_{L}^{{}^{\circ}}f_{L-1}^{{}^{\circ}}\ldots^{{}^{\circ}}f_{1}\left(E\right) \end{array}$$

where $f_{L}^{{}^{\circ}}f_{L-1}^{{}^{\circ}}=f_{L}\left(f_{L-1}\left(.\right)\right)$. For the layer i of the given Attention Net, *f*_*i*_(.) is calculated as follows:

(4) $$\begin{array}{l} f_{i}\left(x\right)=\sigma \left(W_{i}^{k}E_{i-1}^{k}+b_{i}^{k}\right) \end{array}$$

where $E_{i-1}^{k}$ is the output of i-1 hidden layer in the *k*th Attention Net. $W_{i}^{k}$ and $b_{i}^{k}$ are trainable parameters of this layer, σ(.) denotes activation function tanh().

The outputs of all shallow Attention Nets were integrated into an attention matrix $A=\left\{p^{k}|k=1,2,\ldots ,d\right\}\in R^{N\times d}$. In order to prevent the saturation of neuron output caused by the excessive absolute value of the weight, the sigmoid function was used to normalize:

(5) $$\begin{array}{l} A^{\prime }=sigmoid\left(A\right) \end{array}$$

Finally, the weighted feature T was the dot product ⊙ of original data X and attention matrix *A*′. The final weighted feature of the multimodal data is represented as follows:

(6) $$\begin{array}{l} T=\left\{T_{g},T_{c},T_{clin}\right\}\in R^{N\times d}=X⊙A^{\prime } \end{array}$$

#### 2.3.2. Affinity Fusion Module

We propose a fusion method for multimodal data based on affinity network. It consists of three main parts: (1) construction of a bipartite graph; (2) one-mode projection of the bipartite graph; and (3) extraction of fusion feature.

**Bipartite Graph:** In order to capture the structured information between patients and multimodal data, we utilized the gene expression data and CNA data to construct a bipartite. According to the previous method (Sun et al., 2018; Chen et al., 2019), the features from gene expression data and CNA data are standardized and further processed into three and five categories in data preprocessing, respectively. Among them, if the feature value is 0, it is regarded as normal expression, otherwise abnormal expression.

**Figure 2:**
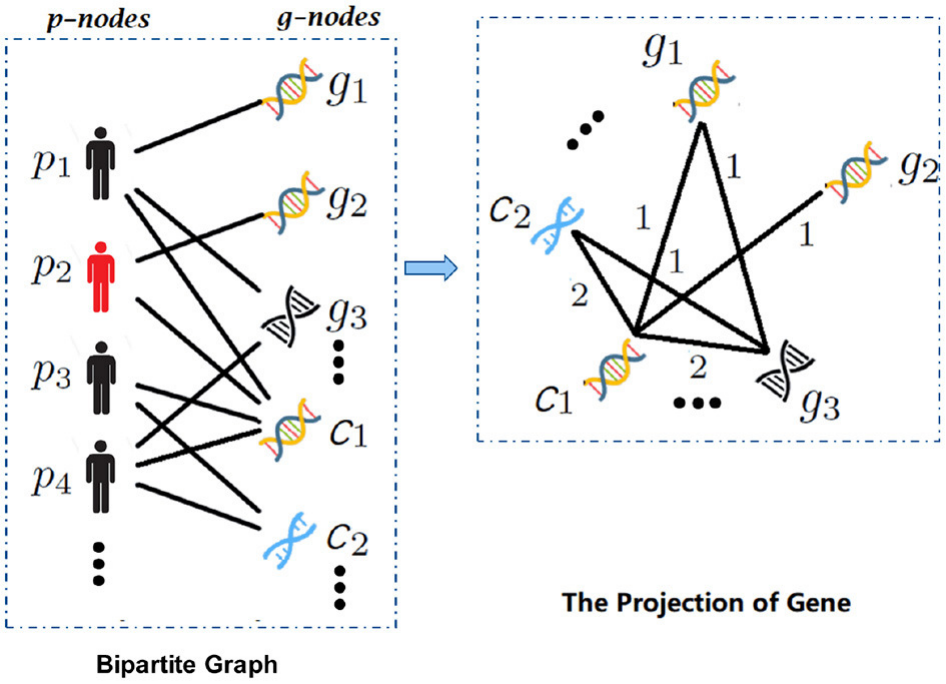
The overview of bipartite graph and one-mode projection. With the input gene expression and copy number alteration (CNA) data, (1) bipartite graph expresses the structured relation between patients and multimodal data, e.g., the edges between patient *p*_*i*_ and *g*_*j*_ (in gene expression data) or *c*_*j*_ (in CNA data); (2) the projection of gene, which establishes the connection of different modalities by one-mode projection.

First, set *G*_*B*_ = (*V, E*) as an undirected bipartite graph. Vertex V consists of two mutually disjoint subsets, namely gene node set and patient node set {p-nodes, g-nodes}. A node in g-nodes represents a feature from gene expression data or CNA data, as shown in the bipartite graph in Figure 2. For each patient, an edge will be built between the patient node and a gene node, only if the gene node value is abnormal expression (non-zero). Finally, we constructed a patient-feature bipartite graph. Obviously, we could intuitively understand gene expression data and CNA data affecting patients from the patient-gene bipartite graph.

In the bipartite graph, the number of the patient nodes is N and the gene nodes is (m+n). Set *B* ∈ [1, 0]^*N*×(*m*+*n*)^ as the bipartite graph relationship matrix, then

(7) $$\begin{array}{l} b_{ij}=\{\begin{array}{l} 1,\;p_{i}g_{j}\in E\\ 0,\;otherwise \end{array} \end{array}$$

where E is a set of edges between p-nodes and g-nodes, *b*_*ij*_ is the element value in B, which indicates the relation between patient i and gene j. Each row of matrix B represents the link relationship of a node in P-nodes, and each column represents the link relationship of a node in g-nodes.

**One-mode projection:** In order to compute the affinity network from multimodal data (establish the connection between different modalities), the bipartite graph relationship matrix B was projected to the g-nodes set through one-mode projection (Le and Pham, 2018). For each patient node *p*_*i*_, we defined a sparse matrix *G*_*i*_ on the vertex set g-nodes. If any two gene nodes have edges with *p*_*i*_, an edge will be built between the two gene nodes. The matrix $G_{i}\in {\left[0,1\right]}^{\left(m+n\right)\times \left(m+n\right)}$ was computed as follows:

(8) $$\begin{array}{l} g_{jk}=\{\begin{array}{l} 1,\;b_{ij}=1\;and\;b_{ik}=1\\ 0,\;otherwise \end{array} \end{array}$$

where *g*_*ik*_ is the element value in *G*_*i*_, which indicates the relation between gene j and k. Then the affinity network G was computed as follows:

(9) $$\begin{array}{l} G=\overset{N}{\underset{i}{\sum }}G_{i} \end{array}$$

where *N* is the number of patient nodes. *G*(*j, k*) indicates the weight between gene j and k.

Further Prune “Weak” Edges: For G, edges with small weights are more likely to be noise. Hence, we pruned “weak” edges by constructing a KNN graph. We defined the affinity matrix *G*′ as follows:

(10) $$\begin{array}{l} G^{\prime }=\psi \left(G,k\right) \end{array}$$

where ψ(., *k*) is the near neighbor chosen function. It keeps the top-k values for each row of a matrix and sets the others to zero.

Normalization: A feasible way is to obtain normalized affinity matrix by degree matrix: $\hat{G^{\prime }}=D^{-1}G^{\prime }$, D is the diagonal matrix whose entries $D\left(i,i\right)=\underset{j}{\sum }G_{ij}^{\prime }$, so that $\underset{j}{\sum }\hat{G_{ij}^{\prime }}=1$. However, this normalization involves self-similarities on the diagonal of $\hat{G^{\prime }}$ matrix, which may lead to numerical instability. One way (Peng et al., 2005) to perform a better normalization is as follows:

(11) $$\begin{array}{l} \hat{G_{ij}^{\prime }}=\{\begin{array}{ll} \frac{G_{ij}^{\prime }}{2\sum_{j\in N_{k}\left(i\right)}G_{ij}^{\prime }}, & j\neq i\\ \frac{1}{2}, & j=i \end{array} \end{array}$$

where *N*_*k*_(*i*) is the indexes of k nearest neighbors of gene i. This normalization method can take out the diagonal self-similarity, and $\underset{j}{\sum }\hat{G_{ij}^{\prime }}=1$ is still valid.

**Extract Fusion Features:** We utilized the affinity matrix to propagate features. Before this, weighted features of the gene expression and CNA modalities were concatenated in the row dimension, each row of which stores features of a sample:

(12) $$\begin{array}{l} Z=\left[T_{G};\;T_{c}\right] \end{array}$$

Inspired by the graph convolutional neural network, we extracted the fusion features by the following formula:

(13) $$\begin{array}{l} F_{f}=f\left(\hat{G^{\prime },Z^{\left(l\right)}}\right)=\sigma \left(Z^{\left(l\right)}\hat{G^{\prime }}W_{f}^{\left(l\right)}\right) \end{array}$$

where *Z*^(0)^ = *Z* and $W_{f}^{\left(l\right)}$ are trainable parameters of l layer, and σ(.) denotes activation function tanh().

#### 2.3.3. DNN Module

In order to compensate the lack of single-modality specific information on fusion features, we utilized DNN module to extract effective features from each modality. The module consists of three deep neural networks. The specific features *F*_*i*_ of each modal were extracted as follows:

(14) $$\begin{array}{l} F_{i}=\sigma \left(W^{\left(l\right)}T_{i}^{\left(l\right)}+b^{l}\right),i\in \left\{g,c,clin\right\} \end{array}$$

where $T_{i}^{\left(0\right)}=T_{i}$, *W*^(*l*)^ is trainable parameters of l layer, *b*^(*l*)^ is the bias vector, σ(.) denotes activation function tanh().

Then, the specific features from DNN module and fusion features from affinity fusion module were concatenated in the row dimension:

(15) $$\begin{array}{l} F=\left[F_{g};F_{c};F_{clin};F_{f}\right] \end{array}$$

Finally, F with multiple fully connected layers was used to predict the survival of breast cancer patients:

(16) $$\begin{array}{l} \hat{y}=sigmoid\left(\sigma \left(W^{\left(l\right)}F^{\left(l\right)}+b^{\left(l\right)}\right)\right) \end{array}$$

where *W*^(*l*)^ and *b*^(*l*)^ are trainable parameters of l layer, σ(.) denotes activation function tanh(), and *F*^(*l*)^ denotes the final multimodal representation at the layer l. Finally, we obtained the final prediction score $\hat{y}$ with sigmoid function.

### 2.4. Optimization

For model optimization, MAFN can be trained with supervised setting. we defined cross entropy loss as objective function. In addition, we used L2 regularization to prevent overfitting. The objective function can be defined as follows:

(17) $$\begin{array}{l} loss\left(X,y,\hat{y}\right)=-\frac{1}{n}\overset{n}{\underset{i}{\sum }}\left[\alpha ylog\left(\hat{y}\right)+\left(1-\alpha \right)\left(1-y\right)log\left(1-\hat{y}\right)\right]\\ \;+\frac{1}{\lambda }\overset{L}{\underset{l=1}{\sum }}∥W_{l}{∥}_{2} \end{array}$$

where y is the real label, $\hat{y}$ is the prediction score, and n is the size of batch. λ and α are the hyperparameters.

## 3. Results and Discussion

### 3.1. Experimental Settings

We implemented our model using Pytorch on a Nvidia GTX 1080 GPU server. The model was trained with Adam optimizer. The learning rate was initialized as e-3, and decayed to e-4 at 6-th epoch. The parameters in section 2.3.1 were set as *E*_*N*_ = 128, *L* = 2, *k* = 10, and *l* = 3. Afterward, The weights between each layer were initialized using normalized initialization proposed by Glorot and Bengio (Glorot and Bengio, 2010). The weights between layers were initialized from a truncated normal distribution defined by:

(18) $$\begin{array}{l} W\sim T\left[-\sqrt{\frac{2}{n_{i}+n_{0}}},\sqrt{\frac{2}{n_{i}+n_{0}}}\right] \end{array}$$

where *n*_*i*_ and *n*_0_ denote the number of input and output of the units, respectively.

### 3.2. Evaluation Metrics

Following (Sun et al., 2007; Arya and Saha, 2020), we adopted AUC as the evaluation metric, which is widely used in survival prediction tasks. We plotted the receiver operating characteristic (ROC) curve to show the interaction between true positive (TP) and false positive (FP). AUC, Accuracy (Acc), Precision (Pre), F1-score, and Recall were also used for performance evaluation. The metrics are evaluated as follows:

(19) $$\begin{array}{l} Accuracy=\frac{TP+TN}{TP+TN+FP+FN} \end{array}$$

(20) $$\begin{array}{l} Precision=\frac{TP}{TP+TN} \end{array}$$

(21) $$\begin{array}{l} Recall=\frac{TP}{TP+FN} \end{array}$$

(22) $$\begin{array}{l} F_{1}=\frac{2Precision\times Recall}{Precision+Recall} \end{array}$$

where TP, TN, FP, and FN stand for true positive, true negative, false positive, and false negative, respectively.

### 3.3. Ablation Study

We conducted ablation studies to validate the effectiveness of two crucial components in our proposed MAFN: attention module and affinity fusion module. We employed the DNN module as our basic network, namely DNN model. Experimental results are shown in Table 3.

**Table 3:** Acc, Pre, F1-score, and Recall predictive performance metrics of multimodal affinity fusion network (MAFN) and single-module models.

**Dataset**	**Methods**	**Acc**	**Pre**	**F1-score**	**Recall**
METABRIC	MAFN(DNN_Affinity_Attention)	0.890	0.905	0.924	0.943
	DNN	0.813	0.858	0.879	0.891
	DNN_Affinity	0.863	0.880	0.907	0.936
	DNN_Attention	0.848	0.884	0.920	0.902
TCGA-BRCA	MAFN(DNN_Affinity_Attention)	0.915	0.921	0.948	0.976
	DNN	0.830	0.847	0.889	0.935
	DNN_Affinity	0.858	0.935	0.906	0.879
	DNN_Attention	0.868	0.900	0.920	0.942
GSE8757	MAFN(DNN_Affinity_Attention)	0.941	0.933	0.824	0.763
	DNN	0.725	0.695	0.548	0.894
	DNN_Affinity	0.941	0.882	0.833	0.789
	DNN_Attention	0.745	0.714	0.566	0.895
GSE69035	MAFN(DNN_Affinity_Attention)	0.876	0.780	0.801	0.810
	DNN	0.775	0.683	0.677	0.808
	DNN_Affinity	0.876	0.800	0.784	0.769
	DNN_Attention	0.764	0.680	0.631	0.692

#### 3.3.1. Validation of the Effectiveness of the Attention and Affinity Fusion Module

**Figure 3:**
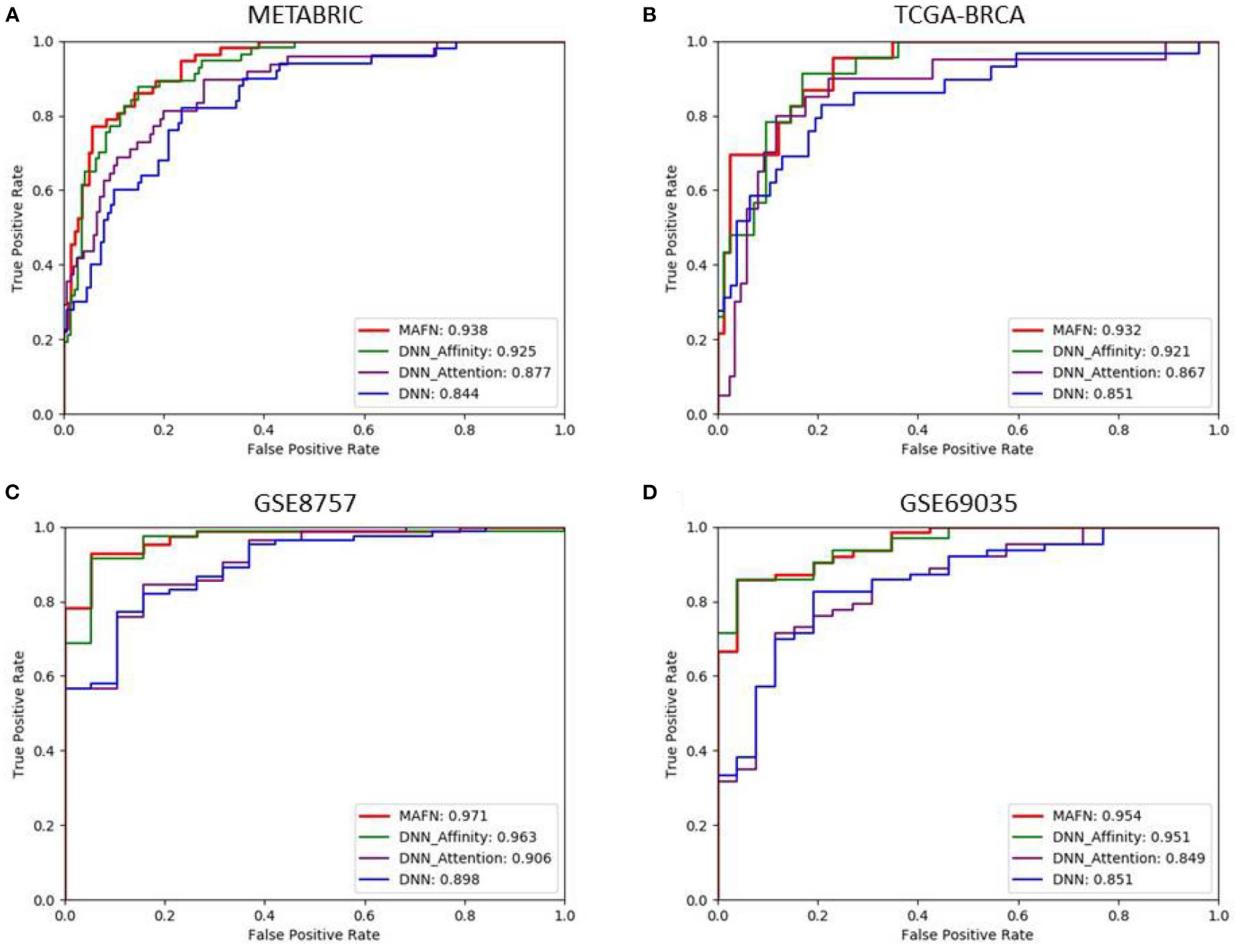
Comparison of ROC curves produced by multimodal affinity fusion network (MAFN) and single module. **(A)** is the result in METABRIC dataset; **(B)** is the result in TCGA-BRCA dataset; **(C)** is the result in GSE8757 dataset; **(D)** is the result in GSE69035 dataset.

1. Evaluation of attention module: To validate the effectiveness of attention module, we compared the performance of DNN model and DNN_Attention model. Figure 3 shows the AUC values of different model. We can find that DNN_Attention achieves consistently better performance than base network on four datasets. For example, the AUC value of DNN_Attention model is improved by 3.3% compared with the DNN model on METABRIC dataset, and 1.6% on TCGA-BRCA dataset. Furthermore, we calculated the corresponding Acc, Pre, F1-score, and Recall of all compared model. In particular, as shown in Table 3, we observed remarkable improvements of 3.5, 2.6, 4.1, and 1.1% for the Acc, Pre, F1-score, and Recall on METABRIC dataset, respectively. These results verify the advantage of using attention module for survival prediction of breast cancer in our proposed MAFN framework by adaptively learning the weight of each feature sequence within multimodal data.
2. Evaluation of affinity fusion module: To validate the effectiveness of our affinity fusion module, we compared the performance of DNN model and DNN_Affinity model. As shown in Figure 3, the AUC value of DNN_Affinity model is improved by 8.1% compared with the DNN model on METABRIC dataset, and 7.0% on TCGA-BRCA dataset. In addition, in terms of other indicators, DNN_Affinity model also achieves corresponding improvement (as shown in Table 2). These results demonstrate that affinity fusion module plays a significant role in compensating for that loss of information of specific features and improving the performance of breast cancer prediction.

Furthermore, we compared the results of MAFN (DNN_Affinity_Attention) model with the model based on a single module improved algorithm (DNN_Affinity and DNN_Attention) on different dataset. As shown in Table 2, the results show that the complementarity of affinity fusion module and attention module.

#### 3.3.2. Validation of the Effectiveness of Multimodal Data

To demonstrate the significance of fusing multimodal data and the effectiveness of affinity fusion module for the prediction of breast cancer survival, we adopted MAFN model to deal with different single types of data (gene expression data or CNA data or clinical data). Furthermore, it can further explore the influence of gene expression data, CNA, and clinical data on breast cancer survival prediction. We designed the following four comparative experiments:

- MAFN with only clinical data.
  In this experiment, we chose only clinical data as input for MAFN model, namely Only_Clin. It is hard to determine outliers in the one-hot coded clinical data. The affinity module cannot construct a bipartite graph based on clinical data. We extracted the features directly by attention module and DNN module.
- MAFN with only gene expression data.
  In this experiment, we chose only gene expression data as input for MAFN model, namely Only_Gene. The affinity fusion module only propagates information in intra-modal of gene expression data.
- MAFN with only CNA data.
  In this experiment, we chose only CNA data as input for MAFN model, namely Only_CNA. The affinity fusion module only propagates information in intra-modal of CNA data.
- MAFN with multimodality data.
  In this experiment, we utilized multimodal data as input for MAFN model, namely MAFN (Gene_CNA_Clin).

**Figure 4:**
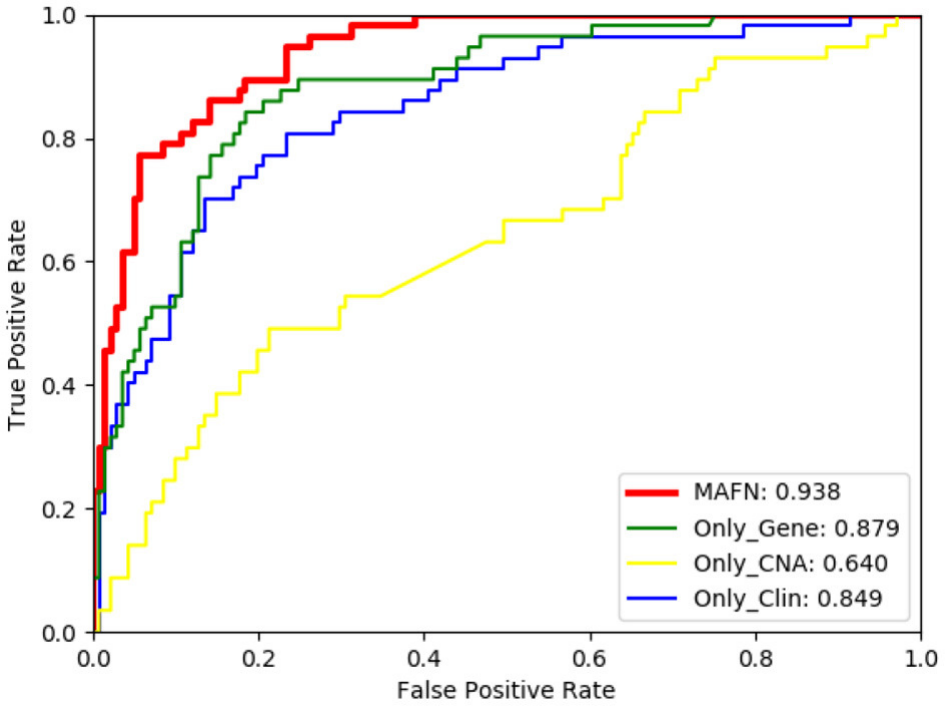
Comparison of ROC curves of multimodal affinity fusion network (MAFN) with different modality data.

**Figure 5:**
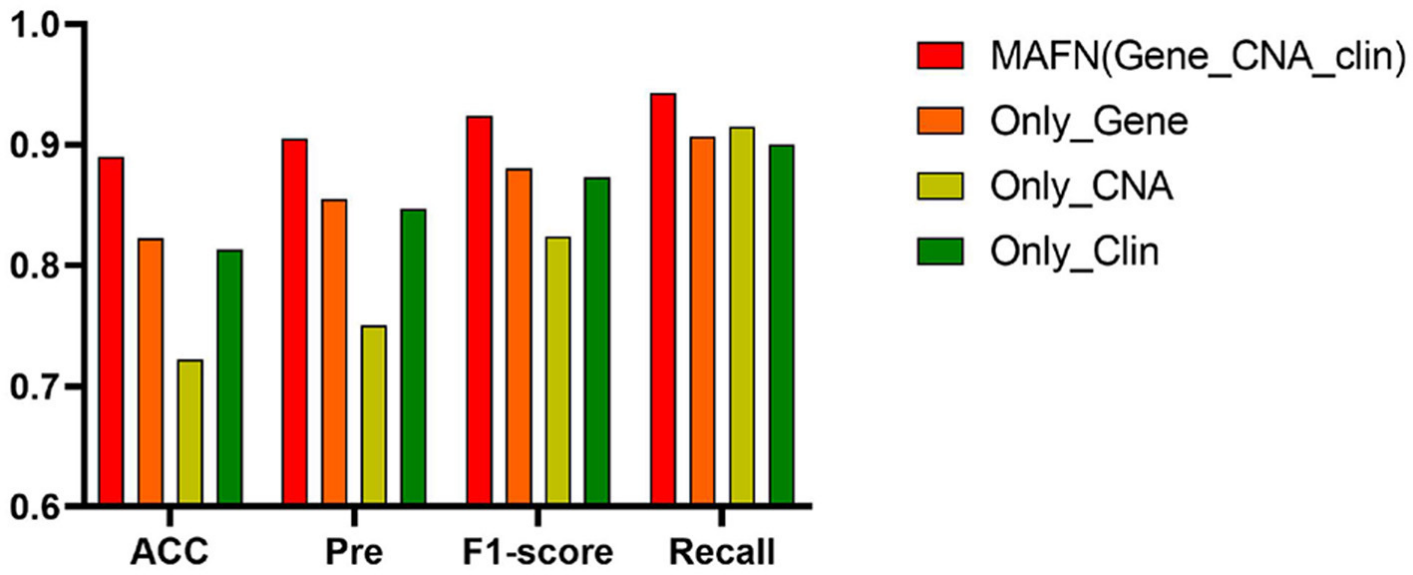
Acc, Pre, F1-score, and Recall of multimodal affinity fusion network (MAFN) with different modality data.

The ROC curves of models using different input on METABEIC dataset are shown in Figure 4. From Figure 4, we observe that compared with using single modality data alone, the application of multimodal data enhances the performance for MAFN. For example, the AUC value of MAFN reaches 93.8%, which is higher than Only_Gene, Only_CNA, and Only_Clin models by 5.9, 29.8, and 8.9%, respectively. In addition, as shown in Figure 5, the Pre of Only_CNA and Only_Clin models are 75.0 and 84.7%, which are lower than Only_Gene model. These results demonstrate that gene expression data yields better classification performance and CNA data and clinical data can provide valuable predictive information additional to those provided by gene expression data. All comparison results confirm the tremendous benefits from integrating multimodal data and features fusion by the Affinity Fusion module in survival prediction. Moreover, we conducted MAFN on METABRIC dataset, in which gene expression data is divided into different expression levels (Jin et al., 2019; Nguyen and Le, 2020; Wei et al., 2020) and detailed information is provided in Supplementary File 1. As shown in Supplementary Table 1, we can find that MAFN is effective in almost common division cases.

### 3.4. Comparison With Other Methods

**Figure 6:**
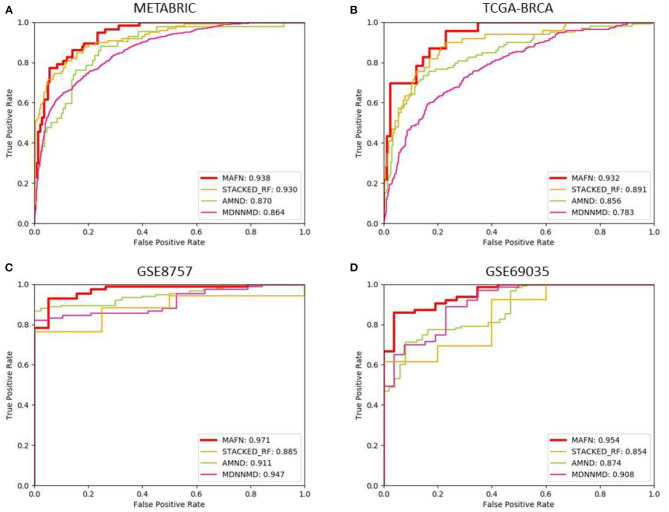
Comparison of ROC curves of multimodal affinity fusion network (MAFN) and existing deep learning-based methods. **(A)** The result in METABRIC dataset; **(B)** the result in TCGA-BRCA dataset; **(C)** the result in GSE8757 dataset; **(D)** the result in GSE69035 dataset.

In order to verify the effect of MAFN, we compared the results of our method with three existing deep learning-based methods, including STACKED_RF (Arya and Saha, 2020), AMND (Chen et al., 2019), and MDNNMD (Sun et al., 2018). Experiments were conducted on METABRIC, TCGA- BRCA, GSE8757, and GSE69035 dataset, and the ROC curves of different methods are plotted in Figure 6. As expected, MAFN achieves better performance among all investigated deep learning-based methods and obtains AUC improvement of 0.8, 6.8, and 7.4% compared with STACKED_RF, AMND, and MDNNMD. From the comparative study presented in Figure 6, we can state that the results on other three dataset are consistent with those on METABRIC dataset. These results show that compared with other methods, MAFN method for multimodal fusion data has remarkable improvements in breast cancer survival prediction.

Additionally, we also analyzed the metrics of Acc, Pre, F1-score, and Recall of different methods. The corresponding results are shown in Table 4. The Acc value of MAFN on METABRIC dataset is 89.0%, which is 4.2, 5.8, and 8.7% higher than those obtained by STACKED_RF, AMND, and MDNNMD, respectively. The results from other three dataset are consistent with those on METABRIC dataset. These results further confirm the effectiveness of MAFN in breast cancer survival prediction.

**Table 4:** Acc, Pre, F1-score, and Recall predictive performance metrics of multimodal affinity fusion network (MAFN) and existing deep learning-based methods.

**Dataset**	**Methods**	**Acc**	**Pre**	**F1-score**	**Recall**
METABRIC	MAFN	0.890	0.905	0.924	0.943
	STACKED_RF	0.902	0.841	0.910	0.923
	AMND	0.848	0.857	0.908	0.972
	MDNNMD	0.832	0.801	0.508	0.372
TCGA-BRCA	MAFN	0.915	0.921	0.948	0.976
	STACKED_RF	0.897	0.913	0.910	0.914
	AMND	0.837	0.851	0.932	0.911
	MDNNMD	0.780	0.765	0.513	0.530
GSE8757	MAFN	0.941	0.933	0.824	0.736
	STACKED_RF	0.879	0.910	0.895	0.903
	AMND	0.814	0.881	0.892	0.886
	MDNNMD	0.868	0.903	0.918	0.933
GSE69035	MAFN	0.876	0.780	0.801	0.810
	STACKED_RF	0.717	0.779	0.856	0.843
	AMND	0.744	0.823	0.816	0.809
	MDNNMD	0.787	0.893	0.840	0.794

To further evaluate the performance of MAFN, we also compared it with three widely used traditional classification methods, including LR (Jefferson et al., 1997), RF (Nguyen et al., 2013), and SVM (Xu et al., 2012). Experiments were conducted on METABRIC and TCGA- BRCA dataset. As shown in Table 5, experimental results show that a more optimal performance was obtained from MAFN compared traditional classification methods. For example, the AUC value of MAFN on TCGA-BRCA dataset is higher than LR, RF, and SVM by 12.1, 19.6, and 21.1%, respectively. At the same time, we could observe that the prediction effect of deep learning method is better than non-deep learning-based methods from Tables 4, 5. Moreover, some researchers (Gao et al., 2019; Poirion et al., 2019; Tran et al., 2020) directly used the survival date as the training label, and also achieved satisfactory results. Since the training target of these methods is inconsistent with MAFN, and thus the comparison experiments cannot be performed.

**Table 5:** AUC, Acc, Pre, F1-score, and Recall predictive performance metrics of MAFN and existing non-deep learning-based methods.

**Dataset**	**Methods**	**AUC**	**Acc**	**Pre**	**F1-score**	**Recall**
METABRIC	MAFN	0.938	0.890	0.905	0.924	0.943
	LR	0.854	0.803	0.847	0.874	0.903
	RF	0.839	0.836	0.841	0.899	0.967
	SVM	0.848	0.818	0.852	0.885	0.920
TCGA-BRCA	MAFN	0.932	0.890	0.905	0.924	0.943
	LR	0.811	0.808	0.879	0.882	0.884
	RF	0.761	0.818	0.816	0.899	0.980
	SVM	0.721	0.776	0.824	0.869	0.919

In conclusion, MAFN is superior to other existing deep learning methods and non-deep learning-based methods on different datasets, indicating that MAFN method has remarkable improvements in breast cancer survival prediction. At the same time, the feasibility of deep neural network with multimodal data fusion and the practicability of multimodal data in the prediction of breast cancer prognosis are further proved.

## 4. Conclusion

In this study, we propose a deep neural network model based on affinity fusion (MAFN) to effectively integrate multimodal data for more accurate breast cancer survival prediction. Our findings suggest that survival prediction methods based fused feature representations from different modalities outperform those using single modality data. Moreover, our proposed attention module and affinity fusion module can efficiently extract more critical information within multimodal data, and capture the structured information within and between the modalities. Meanwhile, DNN module can compensate the lacked single-modality specific information on fusion features. The comprehensive experimental results show that by using fusion features and specific features as input, MAFN compares favorably with the existing methods. The important success of this work is the improvements for the understanding of breast cancer multimodal data fusion and the development of relevant prediction methods for survival. Moreover, this method can be extended to predict the survival time of other similar diseases, providing a new strategy for cancer prognosis.

## Data Availability Statement

The original contributions presented in the study are included in the article/Supplementary Material, further inquiries can be directed to the corresponding author/s.

## Author Contributions

WG conceived and designed the algorithm and analysis, conducted the experiments, and wrote the manuscript. WL and QD performed the biological analysis and wrote the manuscript. QD and XZ provided the research guide. XZ supervised this project. All authors contributed to the article and approved the submitted version.

## Conflict of Interest

The authors declare that the research was conducted in the absence of any commercial or financial relationships that could be construed as a potential conflict of interest.

## Publisher's Note

All claims expressed in this article are solely those of the authors and do not necessarily represent those of their affiliated organizations, or those of the publisher, the editors and the reviewers. Any product that may be evaluated in this article, or claim that may be made by its manufacturer, is not guaranteed or endorsed by the publisher.
