# Supplementary material for: A Multimodal Affinity Fusion Network for Predicting the Survival of Breast Cancer Patients
Source: Front Genet. 2021 Aug 20;12:709027. doi: 10.3389/fgene.2021.709027 (PMC8417828; doi:10.3389/fgene.2021.709027)
Supplement: Supplementary file 1 [file Data_Sheet_1.PDF]

Supplementary File S1

Many studies Jin et al. (2019); Nguyen and Le (2020); Wei et al. (2020) also turned gene expression data into a binary form by the mean/median expression levels over the patients. In order to further explore the effectiveness of MAFN in common division cases, we tried to divide gene expression data into different expression levels. We have conducted two contrast experiments on METABRIC dataset.

(1) MAFN with only binary gene expression data

In this experiment, we chose only binary gene expression data as input for MAFN model, namely Only_Gene_Binary. Affinity Fusion module only propagates information in intra-modal of gene expression data.

(2) MAFN with only ternary gene expression data

In this experiment, we chose only ternary gene expression data as input for MAFN model, namely Only_Gene_Ternary. Affinity Fusion module only propagates information in intra-modal of gene expression data.

Table S1. AUC, ACC, Pre, F1-score, and Recall predictive performance metrics of MAFN using different division methods

Dataset	Methods	AUC	Acc	Pre	F1-score	Recall
METABRIC	Only_Gene_Binary	0.880	0.803	0.831	0.867	0.908
	Only_Gene_Ternary	0.879	0.823	0.855	0.880	0.907

The corresponding results of models using different input on METABEIC dataset are shown in Table S1. From the comparative study presented in Table 3, the AUC value of the MAFN using binary gene expression data is higher than that using ternary gene expression data by 0.1%. At the same time, starting from other indicators, the performance of MAFN using ternary gene expression data is better. These results show that MAFN is effective in common division cases.

REFERENCES

Jin, H., Huang, X., Shao, K., Li, G., Wang, J., Yang, H., et al. (2019). Integrated bioinformatics analysis to identify 15 hub genes in breast cancer. *Oncology letters* 18, 1023–1034

Nguyen, Q.-H. and Le, D.-H. (2020). Improving existing analysis pipeline to identify and analyze cancer driver genes using multi-omics data. *Scientific reports* 10, 1–14

Wei, J., Yin, Y., Deng, Q., Zhou, J., Wang, Y., Yin, G., et al. (2020). Integrative analysis of microrna and gene interactions for revealing candidate signatures in prostate cancer. *Frontiers in Genetics* 11, 176. doi:10.3389/fgene.2020.00176
